# Alterations in intestinal microbiota composition coincide with impaired intestinal morphology and dysfunctional ileal immune response in growing-finishing pigs under constant chronic heat stress

**DOI:** 10.1186/s40104-021-00651-6

**Published:** 2022-01-05

**Authors:** Yunxia Xiong, Shuting Cao, Hao Xiao, Qiwen Wu, Hongbo Yi, Zongyong Jiang, Li Wang

**Affiliations:** State Key Laboratory of Livestock and Poultry Breeding, Key Laboratory of Animal Nutrition and Feed Science in South China Ministry of Agriculture, Maoming Branch, Guangdong Laboratory for Lingnan Modern Agriculture, Guangdong Key Laboratory of Animal Breeding and Nutrition, Institute of Animal Science, Guangdong Academy of Agricultural Sciences, Guangzhou, 510640 China

**Keywords:** Constant chronic heat stress, Growing-finishing pigs, Ileal immune response, Intestinal microbiota, Performance

## Abstract

**Background:**

Previous studies had shown that short-term acute heat stress (HS) affected the host’s metabolism and intestinal microbiota independent of feed intake (FI) reduction, and long-term calorie restriction caused intestinal morphological injuries and gut microbial alterations. However, research on the effects of constant chronic HS on intestinal microbial composition and the roles of FI reduction played in is limited. This study aimed to investigate the effects of 7-day constant chronic HS on the composition of intestinal microbes in growing-finishing pigs, and its relationship with pigs’ performance, intestinal morphology, and ileal immune response. Twenty-four growing-finishing pigs (Duroc × Large White × Landrace, 30 ± 1 kg body weight) were randomly assigned to three treatments (*n* = 8), 1) thermal neutral (TN) conditions (25 ± 1 °C) with ad libitum FI, 2) HS conditions (35 ± 1 °C) with ad libitum FI, 3) pair-fed (PF) with HS under TN conditions to discriminate the confounding effects of dissimilar FI, and the FI was the previous day’s average FI of HS. The small intestinal segments (duodenum, jejunum, and ileum) and feces were collected on d 8.

**Results:**

Results indicated that HS drastically declined (*P* < 0.05) average daily gain (ADG) and average daily feed intake (ADFI) (about 61%) in comparison with TN, and caused hyperpyrexia, meanwhile PF caused hypothermia. Morphological observation by light and electron microscopes showed that both HS and PF treatment decreased (*P *< 0.05) the villus and microvillus height compared with TN. Additionally, HS increased (*P* < 0.05) protein expression of heat shock protein 70 in the duodenum, jejunum, and ileum. Furthermore, the expression of tight junction protein zonula occluden-1 (ZO-1) in the duodenum and ileum, and Occludin in the ileum were enhanced (*P* < 0.05) compared with TN and PF. Moreover, HS significantly enhanced (*P* < 0.05) the mRNA relative expression of inflammatory cytokines (*TLR-2, TLR-4, *and tumor necrosis factor-α* (TNF-α), IL-6, IL-8, PG1–5,* β-defensin 2* (pBD-2)*), mucins (mucin-1 and mucin-2) and P65 protein level in the ileal mucosa tissue. Intestinal microbiota analysis by 16S rRNA sequencing showed lower (*P* < 0.10) *α* diversity in both HS and PF, and a separated cluster of *β* diversity among groups. Compared with TN, HS but not PF mainly reduced (*FDR* < 0.05) Bacteroidetes (phylum), Bacteroidia (class) and elevated the proportions of Proteobacteria (phylum, *FDR* < 0.05), Bacillales (order, *FDR* < 0.05), Planococcaceae (family, *FDR* < 0.05), *Kurthia* (genus, *FDR* < 0.05), Streptococcaceae (family, *FDR* < 0.10) and *Streptococcus* (genus, *FDR* < 0.10). Notably, Lactobacillales (order) was decreased (*FDR* < 0.05) by PF alone. Furthermore, the Spearman correlation analysis indicated that the microbes prevalent in HS were positively (*P* < 0.05) associated with intestinal morphological injuries indicators and ileal immune response parameters, and the microbes reduced in HS were negatively (*P* < 0.05) with the performance data.

**Conclusions:**

Intestinal morphological injuries and ileal immune response caused by constant chronic HS independent of FI showed close connections with alterations in intestinal microbiota in growing-finishing pigs.

## Background

Due to the global warming and intensively reared, heat stress (HS) has been considered as a common challenge to livestock industries for detrimental to animal performance, health, and welfare, mainly manifest in physical disorders, feed intake (FI) reduction, growth retardation, and if severe and prolonged enough even death [1–5]. Growing-finishing pigs are particularly susceptible to heat stress because of few functional sweat glands, thick layer of fat, and high metabolic heat production [6–14]. The intestine is susceptible to the high ambient environment, and HS can cause the increase of intestinal permeability and together with bacterial translocation, which will provoke the immune response via toll-like receptors pathway [15–17]. Especially, ileum was reported more sensitive to HS than other intestinal sections [10]. Gut microbiota changes induced by HS were speculated to activate TLR4/NF-κB signaling pathway contributing to inflammatory response in the colon of pigs [18]. However, the research about the ileal immune response and the associations with gut microbiota alterations is limited.

Intestine is colonized with commensal and pathogenic bacteria, and gut microbial composition can be affected by psychological, environmental, and physical stressors, and associated with metabolic, inflammatory, and infectious diseases [19, 20]. HS was found to alter gut microbial composition in broilers [21, 22], laying hens [23, 24], ducks [25], goats [26], and pigs [27–29]. Our previous research indicated that changes in gut microbiota caused by 24-h acute HS showed interactions with the host’s metabolism [28]. The negative consequences of HS during short term are usually presumed to be related to heat load and duration [4, 10, 27]. However, the alterations in the intestinal microbiota of growing-finishing pigs under constant chronic HS are still unclear.

To less heat production, FI was usually declined by HS, and FI reduction caused by HS was usually thought to be an indirect reason for morphology injuries, and alterations in gut microbiota. Numbers of studies have shown that long-term calorie restriction alone can also affect the intestinal epithelium integrity and gut microbiota [10, 30, 31]. Our previous study revealed that 24-h acute HS affects the gut microbiota independent of FI which may be because of too short duration [28]. Are the alterations in gut microbiota under longer time constant chronic HS partly associated with long-term FI reduction? Whereas there are few studies conducted to assess the confounding effects of constant HS via reduced FI on gut microbiota. In this study, we set a pair-fed (PF) group, which aims to discriminate the confounding effects of dissimilar FI.

This study was conducted to assess the effects of 7-day constant chronic HS on the performance, small intestinal morphology, ileal immune response, and gut microbiota, and aimed to reveal the possible link between alterations in gut microbiota and FI, intestinal morphological injuries, and ileal immune response using a pair-feeding technique.

## Materials and methods

### Animal management and experimental design

The treatments were similar as described before [28]. Briefly, a total of 24 ear-tagged pigs (Duroc × Large White × Landrace, 30 ± 1 kg body weight) housed in constant climate-controlled individual pens were randomly assigned to three treatments (*n* = 8), 1) thermal neutral (TN) conditions (25 ± 1 °C) with ad libitum FI, 2) HS conditions (35 ± 1 °C) with ad libitum FI, 3) pair-fed (PF) with HS under TN conditions to discriminate the confounding effects of dissimilar FI, and the FI was the previous day’s average FI of HS. The relative humidity was maintained at 75 ~ 85% and the treatments lasted for 7 d. Before the trial, all pigs were adapted to TN conditions for one week. Body weight was recorded at the beginning of the experiment and immediately preceding the sacrifice. The pigs were individually fed third a day, and the FI of each pig was recorded. At the end of the experiment, the performance parameters (average daily gain, average daily feed intake, feed: gain ratio) were calculated. Fresh water was added into a graduated water bucket in every morning during the whole experiment, and daily water intake was calculated based on the amount of water added and residue. The rectal temperature was determined by a mercury thermometer. The body surface temperature was measured by a handheld infrared digital thermometer. All pigs received the same basal diet (Table 1) according to the NRC (2012) standard, and provided water ad libitum, and were routinely immunized and health-care according to the requirements. The pig house and metabolic cage were thoroughly disinfected before the test. The test was carried out in the metabolic room of the Institute of Animal Science of Guangdong Academy of Agricultural Sciences.

**Table 1:** Ingredients and nutrient levels of the basal diet (air-dry basis)

Ingredients	Content, %	Nutrient levels^b^	Content
Corn	78.48	ME, MJ/kg	13.81
Soybean meal	11.30	CP, %	13.20
Wheat bran	6.50	Ca, %	0.56
CaHPO_4_	0.57	TP, %	0.44
Limestone	0.99	AP, %	0.24
NaCl	0.40	Lys SID, %	0.85
Cholinechloride (50%)	0.14	Met + Cys SID, %	0.48
*L*-Lys	0.47	Thr SID, %	0.52
*DL*-Met	0.05	Trp SID, %	0.15
*L*-Thr	0.08		
*L*-Trp	0.02		
Premix^a^	1.00		
Total	100		

^a^ The premix provided the following per kilogram of diet: VA 7750 IU, VD_3_ 1750 IU, VE 19 IU, VK 3 mg, VB_12_ 25 μg, VB_1_ 1.9 mg, VB_2_ 6 mg, niacin 25 mg, D-pantothenate 9 mg, folic acid 0.6 mg, VB_6_ 5 mg, biotin 0.05 mg, FeSO_4_•H_2_O 72 mg, CuSO_4_•5H_2_O 10 mg, MnSO_4_•H_2_O 42 mg, ZnSO_4_•H_2_O 72 mg, CaI_2_O_6_ 0.42 mg, Na_2_SeO_3_ 0.29 mg.

^b^ Nutrient levels were calculated values

### Sample collection

 As the experiment finished, after fasting overnight, all pigs were slaughtered. The intestinal tissue was separated and divided into the duodenum, jejunum, and ileum, and placed on the surface of icebox for later use. The tissue of the proximal duodenum, middle jejunum, and distal ileum were annulus sampled. Dissected intestinal sections were opened and washed thoroughly with cold sterile phosphate-buffered saline. Mucosa sample was scraped from the intestinal inner side with a sterile slide. Fresh fecal samples from the rectum were collected into sterilized tubes. All samples were collected and immediately transferred into liquid nitrogen and stored at − 80 °C until subsequent analysis. Annulus small intestine sections samples of about 1.5 cm thickness were placed directly in 4% paraformaldehyde for overnight fixation at room temperature for subsequent H&E staining, and samples about 1 mm thickness were fixed for 1 h in cold 1% osmium tetroxide incacodylate buffer for electron microscopy examination.

### Digesta pH measurement

After each intestinal segment was separated, both ends were tied with sterile cotton thread, then an incision was cut in the middle part, after that a hand-held portable pH digital acidity meter (testo 205, Testo, Lenzkirch, Germany) was inserted into the digestive tract to determine the pH value of content.

### Morphological examination

After dehydration, paraffin embedding, sectioning, dewaxing, hematoxylin-eosin (H&E) staining, dehydration, and sealing, the sample pieces were obtained. Nikon Eclipse E100 microscope equipped with Nikon DS-U3 imaging system (Nikon, Tokyo, Japan) was used to collect images, and Case Viewer software (3DHISTRCH Ltd., Budapest, Hungary) was used to measure intestinal villus height and crypt depth. At least five well-oriented villi were calculated and reported as one number per pig.

### Electron microscopy examination

Tissue sections were fixed for 1 h in 1% cold osmium tetroxide incacodylate buffer. Preparations were fixed by 2.5% glutaral-paraformaldehyde mixture. After dehydration, infiltration, embedding, sectioning, and double staining with uranium acetate saturated lead citrate, ultrathin sections were obtained, and the images were observed by transmission electron microscope (HT7700, Hitachi, Tokyo, Japan). The microvillus height was measured by Adobe Photoshop CS5 software (Adobe, San Jose, CA, USA) with ten microvilli calculated as one number per pig.

### Western blotting

Tissue samples were ground into powder in liquid nitrogen. About 0.1 g sample powder was lysed in 0.9 mL radioimmunoprecipitation assay (RIPA) lysis buffer (Biosharp, Hefei, China) supplemented with 1% protease inhibitors (Biosharp, Hefei, China) for 30 min on ice. After centrifuged at 12,000 r/min at 4 °C for 15 min, the supernatants were collected for western blotting. Protein concentrations of supernatant were evaluated by BCA protein assay kit (Thermo Fisher Scientific, Waltham, MA, USA) according to the manufacturer’s instructions. After adjusted to equal protein concentration with RIPA lysis buffer (Biosharp, Hefei, China), samples were added into 5 × SDS loading buffer (Bio-Rad, Hercules, CA, USA) at the ratio of 4:1, and then heated at 99.0 °C for 10 min. An equal amount of protein (20 μg) was separated by 10% SDS-PAGE gel (Bio-Rad, Hercules, CA, USA) and transferred on 4.5 μm polyvinylidene difluoride (PVDF) membranes (Millipore, Billerica, MA, USA). The membranes were blocked for 1 h with 5% BSA prepared in Tris-buffered saline containing Tween-20 buffer (TBST) at room temperature, and incubated overnight (14–16 h) at 4 °C with primary antibodies in different dilution, followed by washing and incubation with secondary antibodies for 1 h at room temperature. The primary antibodies and the dilution used in this experiment were as follows: rabbit anti-claudin 1(1:1000), rabbit anti-P65 (1:10,000), and mouse anti-heat shock protein 70 (HSP 70, 1:1000) were purchased from Abcam (Cambridge, USA), rabbit anti-Occludin (1:1000) and rabbit anti-zonula occluden-1(ZO-1, 1:250) were obtained from Invitrogen (Carlsbad, CA, USA), and the internal control antibody in this study was mouse anti-β-actin (1:1000) from Abbkine Scientific Co., Ltd. (Wuhan, China). Peroxidase-conjugated goat anti-rabbit IgG (1:50,000) and peroxidase-conjugated goat anti-mouse IgG (1:50,000) were also obtained from Abbkine Scientific Co., Ltd. (Wuhan, China). The protein bands were visualized by chemiluminescence substrate ECL Plus™ Western Blotting Substrate (Thermo Fisher Scientific, Waltham, MA, USA) with a ChemiDoc XRS imaging system (Bio-Rad, Hercules, CA, USA). The optical density of each band was measured using ImageJ software (National Institutes of Health, Bethesda, MD, USA).

### Real-time PCR

Total RNA of tissue samples was extracted with TRIzol reagent (Invitrogen, Carlsbad, CA, USA) according to the manufacturer’s instructions. After assessing the concentrations and the purity with a NanoDrop ND-1000 Spectrophotometer (Nano-Drop Technologies, Wilmington, DE, USA), RNA integrity was verified by visualization of the 18S and 28S ribosomal bands by 1% agarose gel electrophoresis. The first-strand cDNA synthesis was performed by reverse transcription from 1 μg total RNA using the Prime Script RT reagent kit with gDNA Eraser (Takara, Tokyo, Japan) using random hexamer primers according to the manufacturer’s instructions. Real-time PCR (Bio-Rad CFX System, Bio-Rad, Hercules, CA, USA) was performed in a final volume of 20 μL containing 2 μL cDNA product (1:9, v/v), 10 μL iTaq Universal SYBR Green PCR Supermix (2 ×, Bio-Rad, Hercules, California, USA), 6.4 μL RNase free water and 0.8 μL (10 μmol/L) forward and reverse primers (Table 2) designed from porcine sequences published in GenBank with Primer Premier 5.0 (Applied Biosystems, Carlsbad, CA, USA). Primers were purchased from Sangon Biotech (Shanghai, China). The PCR cycling conditions were as follows: initial denaturation program (95 °C for 30 s), amplification and qualification program repeated for 40 cycles (95 °C for 15 s, 60 °C for 30 s, 72 °C for 30 s with a single fluorescence reading). At the end of the PCR running, melt curve analysis was conducted to validate the specificity of the primers, and all determinations were performed in triplicate. The relative expression of target genes to β-actin was quantified using the 2^-ΔΔCT^ method. ΔΔCT is the result of subtracting each group [CT _(target gene)_ – CT _(*β*-actin)_] from TN [CT _(target gene)_ – CT _(*β*-actin)_]. Data were then expressed as fold-changes over values of TN.

**Table 2:** Sequences of primers

Gene	Accession No.	Primer sequence (5′to 3′)	Product size, bp	Annealing temperature, °C
β-Actin	XM_003124280.4	F: CACGCCATCCTGCGTCTGGA R: AGCACCGTGTTGGCGTAGAG	380	60
*TLR-2*	NM_213761.1	F: TCACTTGTCTAACTTATCATCCTCTTG R: TCAGCGAAGGTGTCATTATTGC	162	60
*TLR-4*	NM_001113039.2	F: GCCATCGCTGCTAACATCATC R: CTCATACTCAAAGATACACCATCGG	108	60
*TNF-α*	NM_214022.1	F: CGTCGCCCACGTTGTAGCCAAT R: GCCCATCTGTCGGCACCACC	128	62
*IL-6*	NM_001252429.1	F: TGGCATCTGCCTTCCCTACC R: CAGAGATTTTGCCGAGGATG	132	60
*IL-8*	NM_213867.1	F: TTCGATGCCAGTGCATAAATA R: CTGTACAACCTTCTGCACCCA	176	60
	NM-001204296.1	F: ACACCCATGGGCGCTATGT R: GCCTGCAGAAACCTGCTCAT	68	55.7
	XM-007465997.1	F: CTGCTCCGGGTCCTGTGGGA R: CCCGCTGGCTGGTGCGATAC	100	59
*PG1–5*	XM_005669497.2	F: GTAGGTTCTGCGTCTGTGTCG R: CAAATCCTTCACCGTCTACCA	166	60
*pBD-2*	AY506573.1	F: CCAGAGGTCCGACCACTACA R: GGTCCCTTCAATCCTGTTGAA	88	59

Abbreviations: *F* Forward, *R* Reverse; *TLR-2*, toll-like receptor 2; *TLR-4*, toll-like receptor 4; *TNF-α*, tumor necrosis factor-α; *IL-6*, interleukin-6; *IL-8*, interleukin-8; *PG1–5*, protegrin 1–5; *pBD-2*, porcine beta-defensin 2

### DNA extraction and 16S rRNA amplification

As previously reported [28], after total genome DNA of feces extracted and assessed, the V3-V4 hypervariable regions of the bacterial 16S rRNA gene were amplified via PCR using the forward primer 341F (5′-CCTAYGGGRBGCASCAG-3′) and the reverse primer 806R(5′-GGACTACHVGGGTWTCTAAT-3′) with a sample-unique barcode. The amplification was carried out in 30 μL reaction system with 15 μL of Phusion® High-Fidelity PCR Master Mix (New England Biolabs, MA, USA), 0.2 μmol/L of forward and reverse primers, and about 10 ng template DNA. Thermal cycling consisted of initial denaturation at 98 °C for 1 min, followed by 30 cycles of denaturation at 98 °C for 10 s, annealing at 50 °C for 30 s, and elongation at 72 °C for 30 s, and then a final extension at 72 °C for 5 min. After quantified, PCR products were mixed at equal density ratios and then purified with Gene JETTM Gel Extraction Kit (Thermo Fisher Scientific, Waltham, MA, USA). Sequencing libraries were generated using Ion Plus Fragment Library Kit 48 rxns (Thermo Fisher Scientific, Waltham, MA, USA) following the manufacturer’s recommendations. The library quality was assessed on the Qubit^@^ 2.0 Fluorometer (Thermo Fisher Scientific, Waltham, MA, USA). At last, the library was sequenced on an Ion S5™ XL platform (Thermo Fisher Scientific, Waltham, MA, USA) and 400 bp/600 bp single-end reads were generated.

### Bioinformatics analysis

As described before [28], after assigned to samples based on unique barcode, raw reads were obtained by cutting off the barcode and primer sequence. Quality control of the raw reads was performed using the software Cutadapt (V1.9.1, http://cutadapt.readthedocs.io/en/stable/). Clean reads were obtained by comparing with the Silva database (https://www.arb-silva.de/) using UCHIME algorithm (http://www.drive5.com/usearch/manual/uchime_algo.html) to detect and removed chimera sequences. Then, sequences were clustered into OTUs (Operational taxonomic units) using Uparse software (Uparse v7.0.1001, http://drive5.com/uparse/). OTUs were identified as sequences with ≥ 97% similarity. The annotation process was performed based on Silva Database (https://www.arb-silva.de/) used Mothur algorithm. The phylogenetic relationship of different OTUs, dominant species in different samples was analyzed with MUSCLE software (Version 3.8.31, http://www.drive5.com/muscle/) [32]. OTUs abundance information were normalized using a standard of sequence number corresponding to the sample with the least sequences. Subsequent analysis was based on the normalized data of OTUs abundance information. Bioinformatics and statistical analyses were performed using the QIIME (Version1.7.0) and R (Version 3.0.3, http://www.r-project.org/). A Venn diagram was generated using the R package ‘Venn Diagram’ to visualize the shared and unique OTUs among groups. Alpha diversity indices (Chao1, Shannon) were applied to analyze the richness and diversity of the sequences, and difference analysis between groups was performed with Wilcox rank-sum test. Beta diversity analysis was used to evaluate the structure and distribution of the microbial genetic communities among the samples, and beta diversity based on both weighted and unweighted Unifrac distance was calculated. ANOSIM test (non-parametric test) was used to detect differences in community structure between groups based on OTUs relative abundance, and generated a test statistic R to assess the difference between and within groups. The R value close to 1 suggests dissimilarity between groups, while the R value close to 0 suggests an even distribution of high and low ranks within and between groups. The reliability of ANOSIM test is represented by *P*-value, and *P* < 0.05 indicates significance. OTUs-based principal component analysis (PCA), unweighted Unifrac distance-based and weighted Unifrac distance-based Principal Coordinate Analysis (PCoA), and Binary-Jaccard based nonmetric multidimensional scaling (NMDS) plot were performed to show the distribution of samples. NMDS plot was conducted with a conventional cut-off of < 0.20 for the stress value. Unweighted Pair-group Method with Arithmetic Means (UPGMA) Clustering was performed as a type of hierarchical clustering method to interpret the distance matrix using average linkage. Linear discriminant analysis effect size (LEfSe) analysis was used test to determine the biomarkers in different groups, and perform LDA scores to estimate the effect size (threshold ≥ 4). The significant differential bacteria of top 10 at each level were determined by Kruskal-Wallis test followed by Dunn test, and false discovery rate (FDR) values were estimated using the Bonferroni method to control for multiple testing. The Spearman correlation analysis was applied to analyze the associations of the significantly differential microbes with the performance, intestinal morphological injuries indicators, and ileal immune response parameters. The significance was tested by corr.test in the psych package from R, after which the visualization work was done by the pheatmap function in pheatmap package.

### Statistical analysis

Data of the performance and physical responses variables, intestinal morphological injuries indicators, and ileal immune response parameters are presented as mean ± standard error (SEM). Analysis was performed using SPSS18.0 Software (IBM Corp, Armonk, NY, USA) and GraphPad Prism Version 5 (GraphPad Software Inc., San Diego, CA, USA). Statistical analysis of the difference was performed by one-way analysis of variance (ANOVA) with Fisher’s least significant difference (LSD) post hoc test. Differences were considered to be significant at *P* < 0.05, and 0.05 ≤ *P* < 0.10 mean a trend.

## Results

### Constant chronic HS decreased the performance and affected normal physiological responses

Because one head of pig in the HS group dead on the 3rd day of experiment, the subsequent analysis of HS group was based on the data of the remaining 7 pigs. Effects of constant chronic HS on the performance and physiological responses of growing-finishing pigs were listed in Table 3. After 7-day constant chronic HS or feed limited, final body weight was declined (*P* < 0.05) compared with TN, and even caused body weight loss (0.70–0.96 kg). HS or PF treatment significantly decreased (*P* < 0.05) ADFI, about 61% reduction compared to TN, as well as water intake, but the ratio of water to feed was increased (*P* < 0.05). Compared with TN, HS significantly elevated (*P* < 0.05) the rectal temperature and body surface temperature (jugular, back, hind leg), while PF significantly decreased (*P* < 0.05) body temperature.

**Table 3:** Effects of constant chronic heat stress on the performance and physiological responses in growing-finishing pigs

Items	Treatments			*P-*value
	TN	HS	PF	
Initial body weight, kg	34.06 ± 0.80	33.99 ± 0.71	34.14 ± 0.90	0.992
Finial body weight, kg	38.92 ± 1.34^a^	33.29 ± 0.56^b^	33.18 ± 0.83^b^	< 0.001
Average daily gain, kg/d	0.79 ± 0.04^a^	−0.10 ± 0.06^b^	− 0.14 ± 0.03^b^	< 0.001
Average daily feed intake, kg/d	1.37 ± 0.08^a^	0.54 ± 0.04^b^	0.51 ± 0.00^b^	< 0.001
Water intake, L/d	6.35 ± 0.36^a^	4.81 ± 0.26^b^	4.32 ± 0.28^b^	< 0.001
Ratio of water to feed	4.76 ± 0.44^b^	9.50 ± 1.38^a^	8.46 ± 0.55^a^	0.002
Rectal temperature, °C	39.5 ± 0.1^b^	41.0 ± 0.1^a^	39.1 ± 0.1^c^	< 0.001
Jugular temperature, °C	39.5 ± 0.1^b^	41.4 ± 0.1^a^	38.4 ± 0.1^c^	< 0.001
Back temperature, °C	39.8 ± 0.1^b^	41.7 ± 0.1^a^	38.7 ± 0.1^c^	< 0.001
Hind leg temperature, °C	39.9 ± 0.1^b^	41.9 ± 0.2^a^	38.8 ± 0.1^c^	< 0.001

*TN*, thermal neutral conditions (25 ± 1 °C); *HS*, heat stress conditions (35 ± 1 °C); *PF*, pair-fed with HS under TN conditions (25 ± 1 °C). Data were expressed as mean ± SEM (*n* = 8 for TN and PF, *n* = 7 for HS). Treatments with different superscript letters were significantly different at *P* < 0.05

### Constant chronic HS decreased pH value in the small intestinal segments

As presented in Table 4, pH value of the digesta in the different small intestine sections (duodenum, jejunum, ileum) was measured. HS significantly decreased (*P* < 0.05) pH value of digesta in the jejunum and ileum compared with PF.

**Table 4:** Effects of constant chronic heat stress on pH value of small intestine segments in growing-finishing pigs

Items	Treatments			*P-*value
	TN	HS	PF	
Duodenum	5.33 ± 0.48	4.96 ± 0.19	4.34 ± 0.62	0.337
Jejunum	6.37 ± 0.14^ab^	6.02 ± 0.20^b^	6.58 ± 0.08^a^	0.042
Ileum	6.58 ± 0.10^ab^	6.21 ± 0.19^b^	6.67 ± 0.10^a^	0.060

*TN*, thermal neutral conditions (25 ± 1 °C); *HS*, heat stress conditions (35 ± 1 °C); *PF*, pair-fed with HS under TN conditions (25 ± 1 °C). Data were expressed as mean ± SEM (*n* = 8 for TN and PF, *n* = 7 for HS). Treatments with different superscript letters were significantly different at *P* < 0.05

### Constant chronic HS caused morphological injuries in the small intestine

**Fig. 1:**
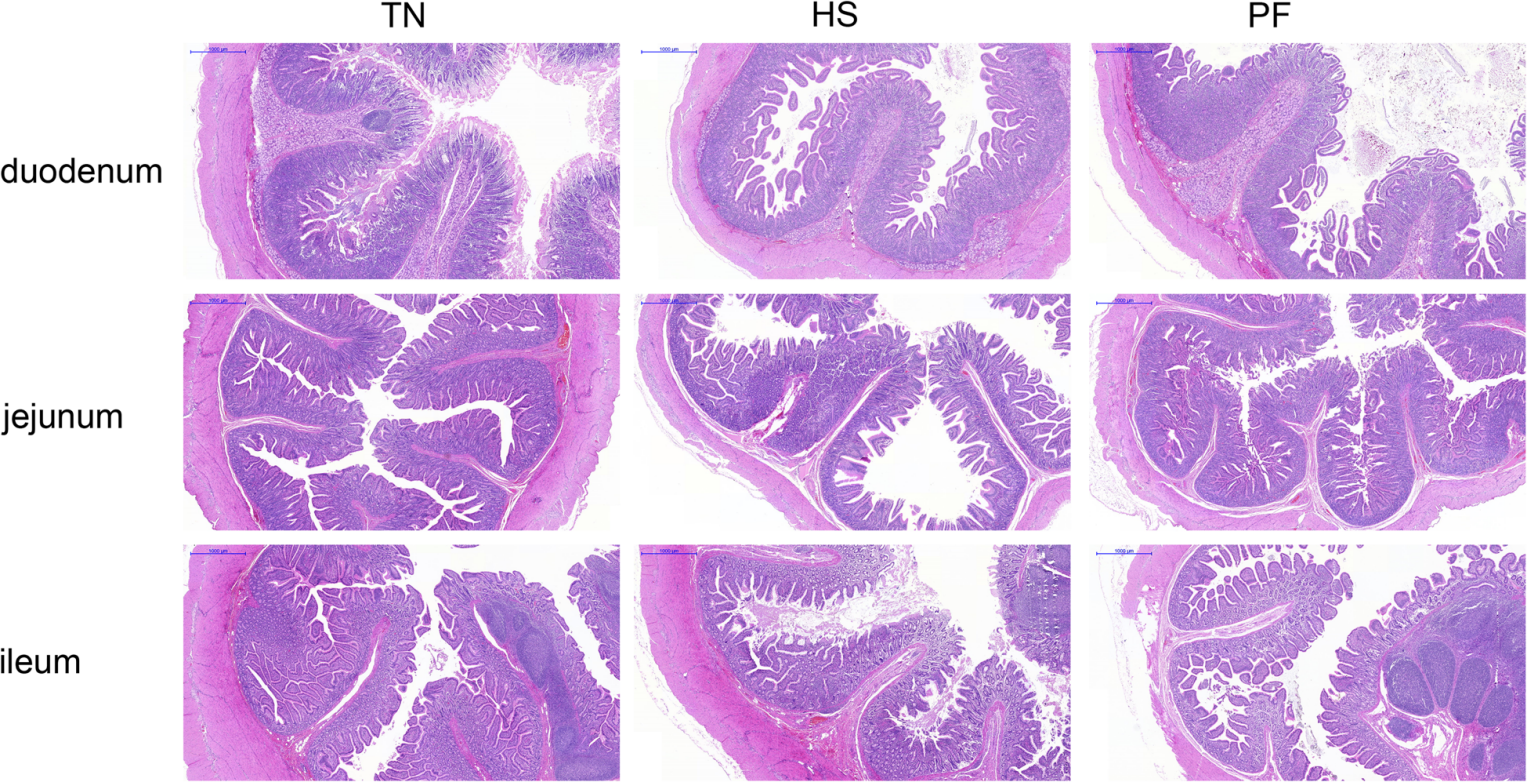
Effects of constant chronic heat stress on morphology of small intestine segments in growing-finishing pigs by H&E-staining (scale bar 1000 μm). TN, thermal neutral conditions (25 ± 1 °C); HS, heat stress conditions (35 ± 1 °C); PF, pair-fed with HS under TN conditions (25 ± 1 °C)

**Fig. 2:**
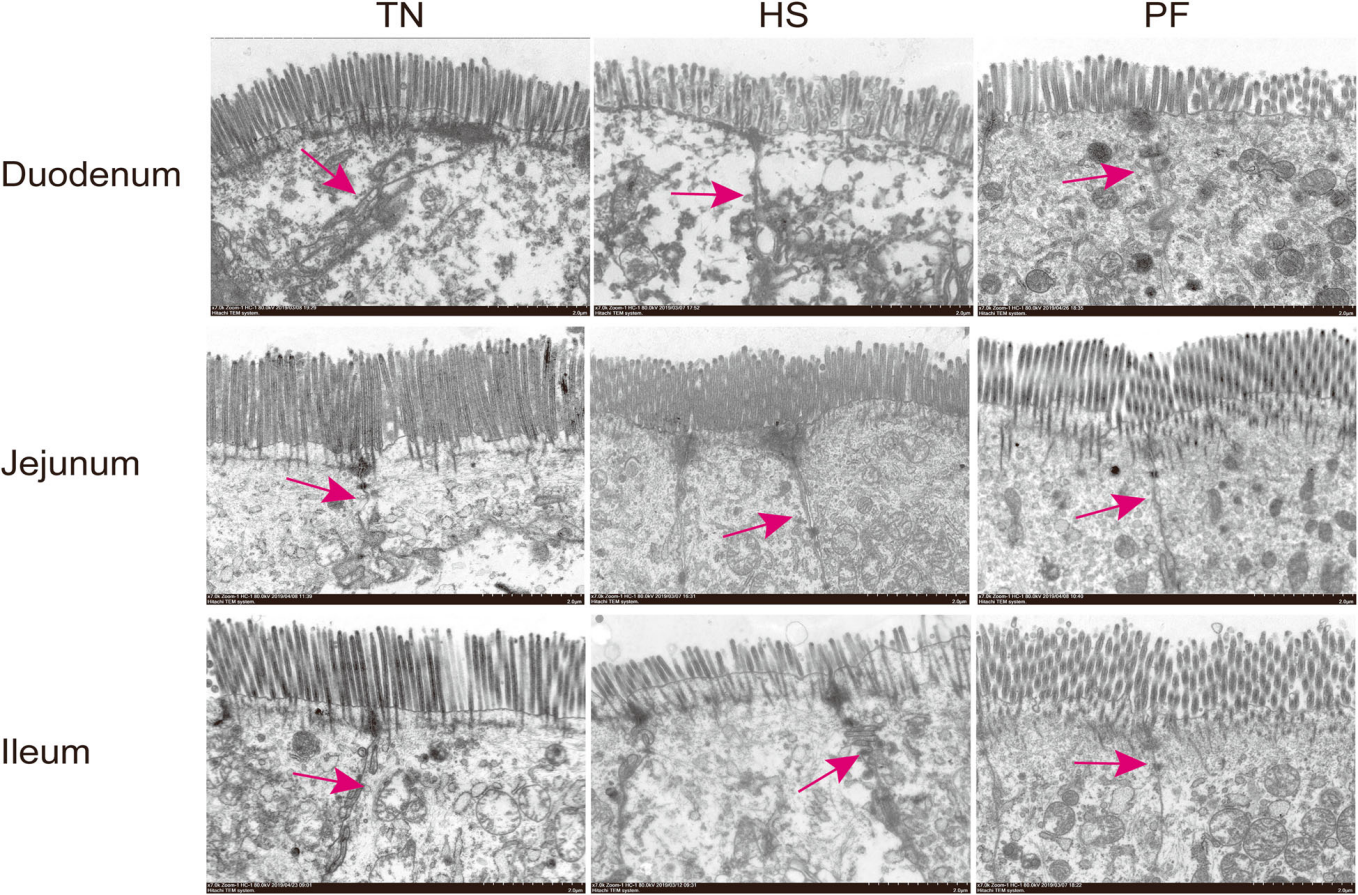
Effects of constant chronic heat stress on the ultrastructure of small intestine segments in growing-finishing pigs by electron microscopy (scale bar 2 μm). The red arrows indicated the location of tight junction structure. TN, thermal neutral conditions (25 ± 1 °C); HS, heat stress conditions (35 ± 1 °C); PF, pair-fed with HS under TN conditions (25 ± 1 °C)

As illustrated in Fig.1 and Fig.2, HS led to marked morphological injuries in the small intestine, such as villi tips desquamation and the lamina propria exposing. To quantify the extent of damage, villus height, microvillus height, and crypt depth were measured. As depicted in Tables 5 and 6, both HS and PF decreased villus and microvillus height (*P* < 0.05) in the duodenum, jejunum, and ileum compared with TN. In addition, HS also significantly decreased (*P* < 0.05) the ratio of villus height to crypt depth in different small intestine sections (duodenum, jejunum, and ileum).

**Table 5:** Effects of constant chronic heat stress on villus height and crypt depth of small intestine segments in growing-finishing pigs

Items	Treatments			*P*-value
	TN	HS	PF	
Duodenum				
Villus height, μm	574.50 ± 25.54^a^	379.49 ± 36.00^b^	414.50 ± 25.26^b^	< 0.001
Crypt depth, μm	505.42 ± 17.15	449.10 ± 38.36	396.94 ± 41.88	0.115
Ratio of villus height to crypt depth	1.14 ± 0.02^a^	0.84 ± 0.03^b^	1.07 ± 0.05^a^	< 0.001
Jejunum				
Villus height, μm	510.37 ± 39.01^a^	319.29 ± 28.62^b^	384.90 ± 11.37^b^	< 0.001
Crypt depth, μm	292.53 ± 9.28^a^	218.69 ± 26.00^b^	218.78 ± 17.76^b^	0.021
Ratio of villus height to crypt depth	1.73 ± 0.08^a^	1.49 ± 0.07^b^	1.80 ± 0.09^a^	0.042
Ileum				
Villus height, μm	526.36 ± 25.43^a^	298.98 ± 28.33^b^	367.27 ± 25.96^b^	< 0.001
Crypt depth, μm	403.73 ± 23.42	367.29 ± 35.48	328.54 ± 30.47	0.244
Ratio of villus height to crypt depth	1.31 ± 0.03^a^	0.82 ± 0.04^c^	1.14 ± 0.06^b^	< 0.001

*TN*, thermal neutral conditions (25 ± 1 °C); *HS*, heat stress conditions (35 ± 1 °C); *PF*, pair-fed with HS with TN conditions (25 ± 1 °C). Data were expressed as mean ± SEM (*n* = 8 for TN and PF, *n* = 7 for HS). Treatments with different superscript letters were significantly different at *P* < 0.05

**Table 6:** Effects of constant chronic heat stress on microvillus height of small intestine segments in growing-finishing pigs, μm

Items	Treatments			*P*-value
	TN	HS	PF	
Duodenum	1.66 ± 0.06^a^	1.42 ± 0.04^b^	1.40 ± 0.09^b^	0.029
Jejunum	2.74 ± 0.07^a^	1.91 ± 0.11^b^	1.94 ± 0.07^b^	< 0.001
Ileum	2.73 ± 0.14^a^	2.01 ± 0.13^b^	2.24 ± 0.12^b^	0.004

*TN*, thermal neutral conditions (25 ± 1 °C); *HS*, heat stress conditions (35 ± 1 °C); *PF*, pair-fed with HS under TN conditions (25 ± 1 °C). Data are expressed as mean ± SEM (*n* = 8 for TN and PF, *n* = 7 for HS). Treatments with different superscript letters were significantly different at *P* < 0.05

### Constant chronic HS elevated the expression of heat shock protein 70 and tight junction proteins

**Fig. 3:**
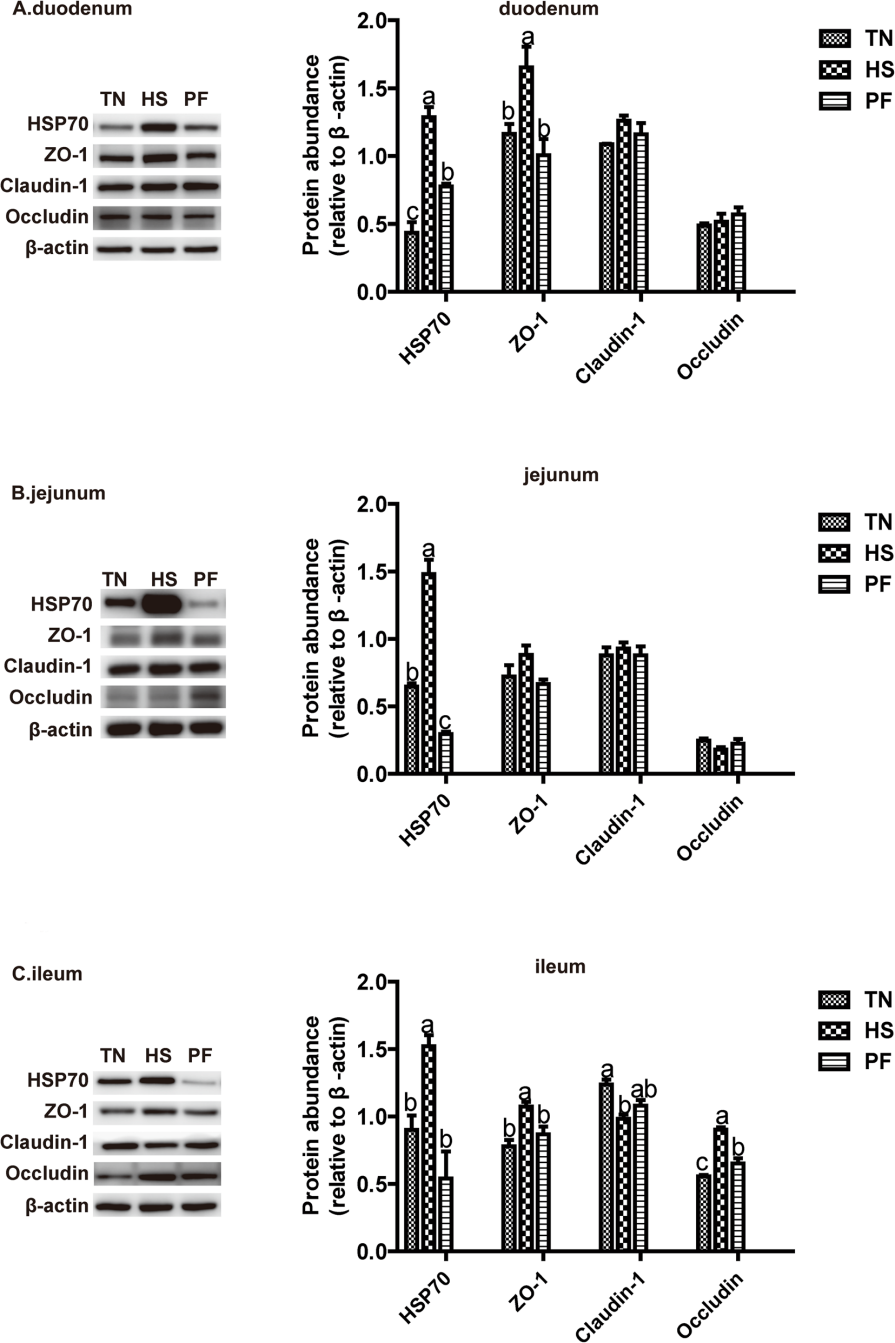
Effects of constant chronic heat stress on the expression of heat shock protein and tight junction proteins of small intestine sections in growing-finishing pigs. A, B, C presented duodenum, jejunum, and ileum respectively. TN, thermal neutral conditions (25 ± 1 °C); HS, heat stress conditions (35 ± 1 °C); PF, pair-fed with HS under TN conditions (25 ± 1 °C). The bar graphs showed the protein band intensity. All data were expressed as the mean ± SEM (*n* = 8 for TN and PF, *n* = 7 for HS). Differences were determined by one-way ANOVA followed by LSD test. Groups without a common letter mean significant differences (*P* < 0.05). Abbreviations: HSP 70, heat shock protein 70, ZO-1, zonula occluden-1

As shown in Fig.3, HS increased (*P* < 0.05) expression of HSP 70 in the duodenum, jejunum, and ileum, and induced up-regulation (*P* < 0.05) of the expression of tight junction protein ZO-1 in the duodenum and ileum, and Occludin in the ileum compared with TN and PF.

### Constant chronic HS activated the immune response in the ileum by NF-κB pathway

**Fig. 4:**
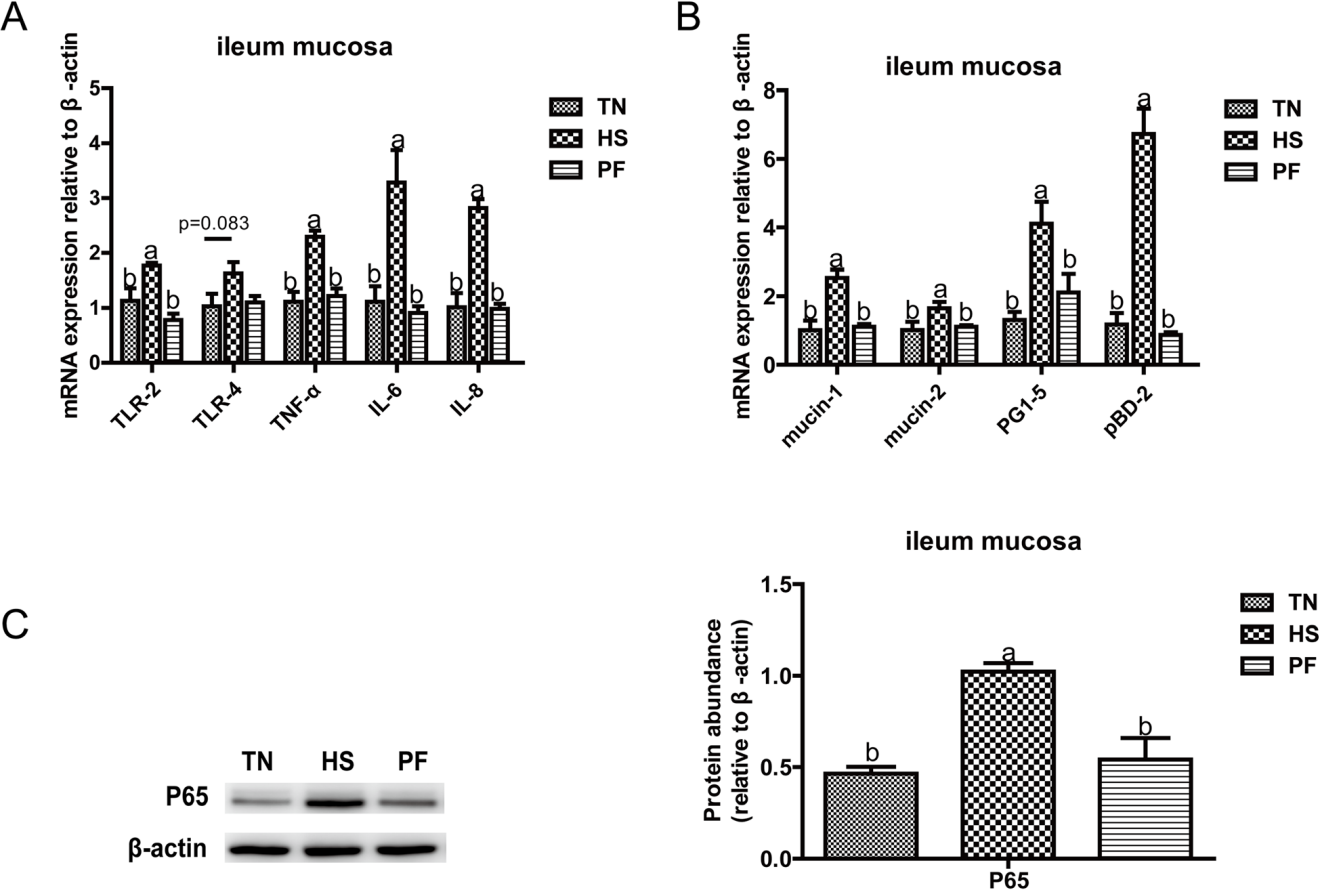
Constant chronic heat stress provokes the immune response in the ileum by NF-κB pathway. Relative mRNA expression levels of *TLR-2* (A), *TLR-4* (A), *TNF-α*(A), *IL-6* (A), *IL-8* (A), *mucin-1*(B), *mucin-2*(B), *PG1–5*(B), pBD2(B) in the ileum of growing-finishing pigs were detected by Real Time-PCR. Protein expression of P65 (C) in the ileum of growing-finish pigs was detected by western blotting and the bar graphs showed the protein band intensity. All data were expressed as the mean ± SEM (*n* = 8 for TN and PF, *n* = 7 for HS). TN, thermal neutral conditions (25 ± 1 °C); HS, heat stress conditions (35 ± 1 °C); PF, pair-fed with HS under TN conditions (25 ± 1 °C). Differences were determined by one-way ANOVA followed by LSD test. Groups without a common letter mean significant differences (*P* < 0.05). Abbreviations: *PG1–5*, protegrin 1–5; *pBD2*, porcine beta-defensin 2; *TLR-2*, toll-like receptor 2; *TLR-4*, toll-like receptor 4; *TNF-α*, tumor necrosis factor-α; *IL-6*, interleukin- 6; *IL-8*, interleukin- 8

Immune-related gene expression of Toll-like receptor 2 (*TLR-2*), *TLR-4*, tumor necrosis factor-α (*TNF-α*), interleukin 6 (*IL-6*)*, IL-8* were found to be up-regulated (*P* < 0.05) by HS compared with TN (Fig.4A). Moreover, the protein expression of P65 was also increased by HS compared with TN (*P* < 0.05). Further, compared with TN, HS elevated (*P* < 0.05) gene expression of mucins (mucin-1, mucin-2) and antimicrobial peptide (protegrin 1–5 [*PG1–5*], porcine beta-defensin 2 [*pBD-2*]) (Fig.4B).

### Constant chronic HS influenced the intestinal microbial composition

**Fig. 5:**
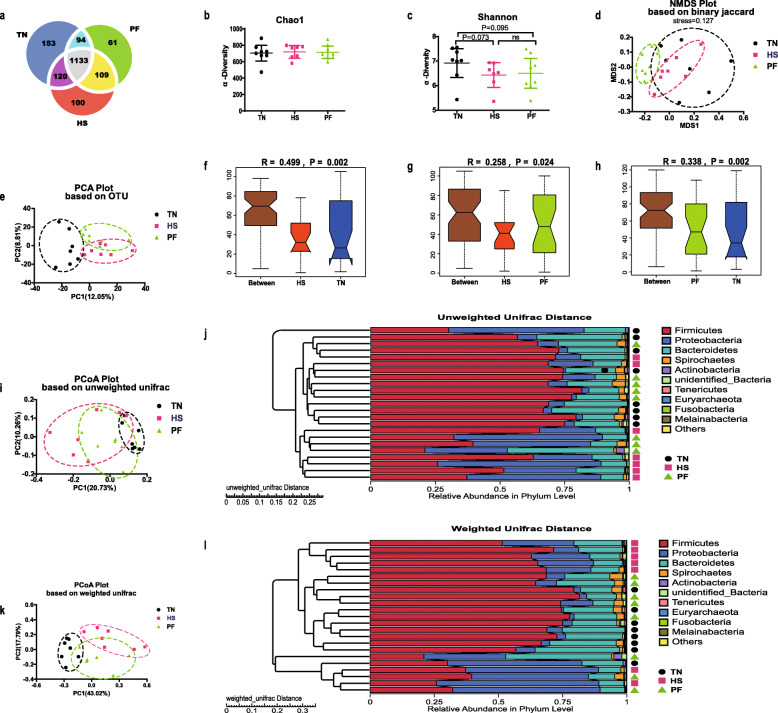
Effects of constant chronic heat stress on intestinal microbial communities. Venn diagram showed the proportion of common and special OTUs among groups (**a**). Alpha diversity index such as Chao1 (**b**) and Shannon (**c**) index indicated the diversity and evenness. Binary-Jaccard distance-based NMDS plot (**d**), OTUs-based PCA plot (**e**), unweighted unifrac distance-based PCoA plot (**i**), weighted unifrac distance-based PCoA plot (**k**) were used to display the distribution of the samples among groups. ANOSIM test was performed to detect differences in community structure between groups based on OTUs relative abundance (**f**, **g**, **h**). UPGMA clustering was conducted based on unweighted unifrac distance and weighted unifrac distance (**j**, **i**). Differences of α-diversity indices were determined by Wilcox rank-sum test, and differences of β-diversity indices were determined by ANOSIM test. * presented *P* < 0.05, *** presented *P* < 0.001, ns mean no significant difference. TN, thermal neutral conditions (25 ± 1 °C); HS, heat stress conditions (35 ± 1 °C); PF, pair-fed with HS under TN conditions (25 ± 1 °C). *n* = 8 for TN and PF, *n* = 7 for HS

After quality control, a total of 1,678,038 clean reads were procured from 23 fecal samples, and clustered into average 755 OTUs per sample, resulting in a total of 1770 OTUs for all samples, and the average sequence effective (the ratio of the number of clean reads to raw reads) was 94.11% per sample. The common and special OTUs proportion among groups was presented in Fig.5a.

**Fig. 6:**
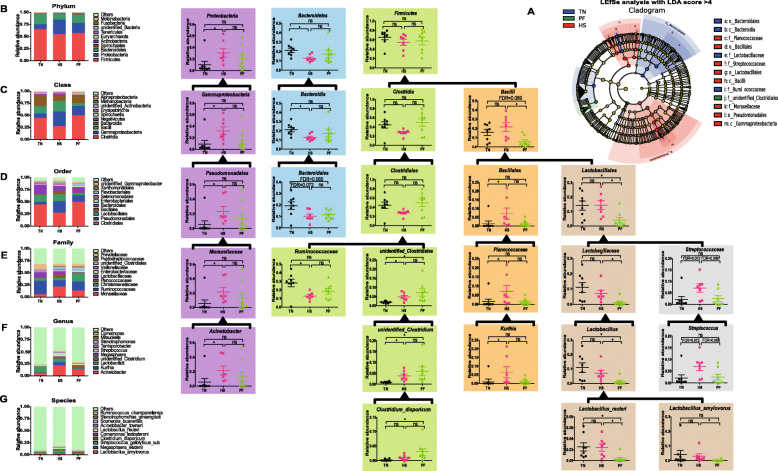
Effects of constant chronic heat stress on the relative abundance of microbial species. The LEfSe analysis (LDA score ≥ 4) identified the biomarker species (**A**). The top 10 phylum, class, order, family, genus, and species and the significantly different microbial at each level were showed (**B**). TN, thermal neutral conditions (25 ± 1 °C); HS, heat stress conditions (35 ± 1 °C); PF, pair-fed with HS under TN conditions (25 ± 1 °C). Brackets indicated the affiliation between species. Differences were determined by Kruskal-Wallis test followed by Dunn test, and false discovery rate (FDR) values were estimated using the Bonferroni method to control for multiple testing (* presented FDR < 0.05, ns mean no significant difference). *n* = 8 for TN and PF, *n* = 7 for HS

There was no difference in Chao1 index among groups (Fig.5b). Differently, both HS and PF exhibited a lower Shannon index (*P* = 0.073, *P* = 0.095) compared with TN (Fig.5c). β diversity analysis was showed in OTUs-based PCA, and weighted and unweighted Unifrac distance-based PCoA plot, which indicated the distribution of the community of samples (Fig.5d, e, i, k), and shown divergence of the community structure among groups. Moreover, Anosim tests confirmed the dissimilarity (*P* < 0.05) community composition between each group (Fig.5f, g, h). LEfSe analysis identified 13 discriminative bacteria among the three groups (Fig.6A). Gammaproteobacteria (class), Bacilli (class), Pseudomonadales (order), Moraxellaceae (family), Lactobacillales (order), Bacillales (order), Planococcaceae (family), and Streptococcaceae (family) were more prevalent in HS. Ruminococcaceae (family), Lactobacillaceae (family), Bacteroidia (class), and Bacteroidales (order) were more abundant in TN. The biomarker microbe in PF was unidentified *Clostridiales* (family).

The composition of top 10 phylum (Fig.6B), class (Fig.6C), order (Fig.6D), family (Fig.6E), genus (Fig.6F), and species (Fig.6G) were provided. Results indicated that Firmicutes (phylum, 54.65–65.49%), Bacteroidetes (phylum, 12.68–21.31%), and Proteobacteria (phylum, 10.00–30.65%) were major phylum (95.53–97.99%) of fecal microbiota in growing-finishing pigs. At genus level, the most prevalent microbes were *Acinetobacter* (5.45–21.65%), *Kurthia* (1.26–6.46%), and *Lactobacillus* (7.05–11.65%)*.* Compared with TN, HS significantly increased (*FDR* < 0.05) the abundance of Proteobacteria (phylum), γ-Proteobacteria (class), Pseudomonadales (order), Moraxellaceae (family) and *Acinetobacter* (genus), and Proteobacteria (phylum) became the second most enriched phylum instead of Bacteroidetes (phylum) in HS. The relative amount of Bacteroidetes (phylum, *FDR* < 0.05), Bacteroidia (class, *FDR* < 0.05), and Bacteroidales (order, *FDR* = 0.072) was decreased by HS. Belonging to Firmicutes (phylum), Clostridia (class), Clostridiales (order), Ruminococcaceae (family) was significantly decreased (*FDR* < 0.05) in HS when compared with TN, while unidentified_Clostridiales (family), and *unidentified_Clostridium* (genus) were increased (*FDR* < 0.05) in PF and HS compared with TN. However, also belonging to Firmicutes (phylum), Bacill (class) was decreased (*FDR* = 0.089, *FDR* < 0.05) in PF compared with TN or HS. Belonging to Bacill (class), the Bacillales (order), and Planococcaceae (family), and *Kurthia* (genus) presented different situations, and all was significant increased (*FDR* < 0.05) in HS independent of FI. Interestingly, HS didn’t affect (*FDR* > 0.05) the relative richness of Lactobacillales (order), Lactobacillaceae (family), *Lactobacillus* (genus), *Lactobacillus reuteri* (species), and *Lactobacillus amylovorus* (species) when compared with TN, but PF decreased (*FDR* < 0.05) the richness of the above microbes in comparison with HS, and decreased (*FDR* < 0.05) the richness of the above microbes except *Lactobacillus amylovorus* (species) in comparison with TN. Furthermore, HS significantly increased (*FDR* < 0.10) the abundance of Streptococcaceae (family) and *Streptococcus* (genus).

### Associations of significantly differential intestinal microbes with the performance, intestinal morphological injuries indicators, and ileal immune response parameters under constant chronic HS

**Fig. 7:**
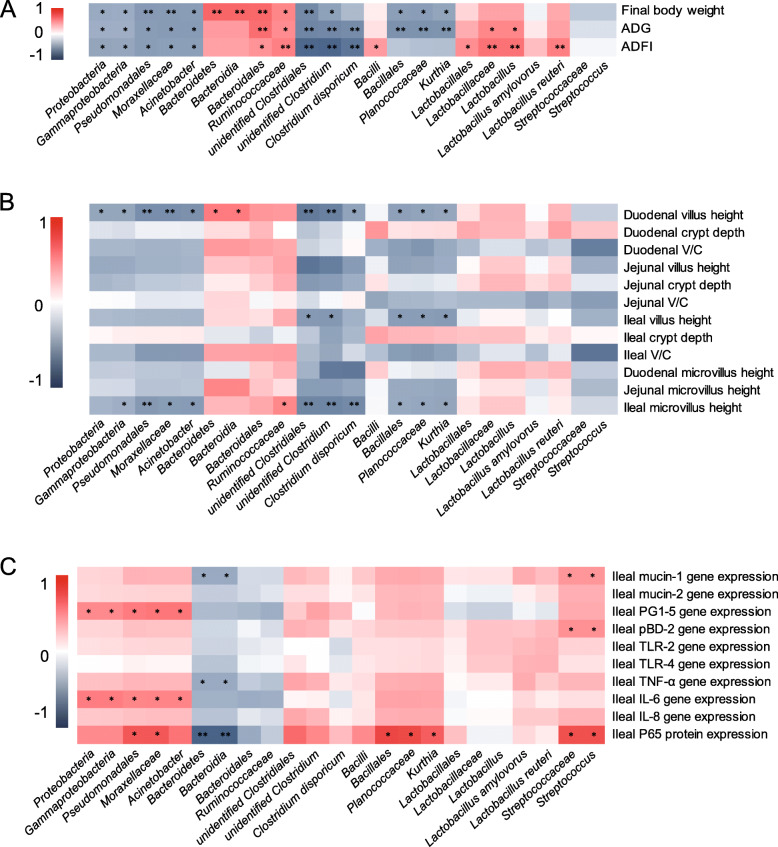
The Spearman correlation analysis between significantly differential microbes and the performance (**A**), small intestinal morphological injuries indicators (**B**), and ileal immune response parameters (**C**) under constant chronic HS. The heatmap of the correlation coefficient, the red represents positive correlation and the blue represents negative correlation, respectively (* presented *P* < 0.05, ** presented *P* < 0.01). Abbreviations: ADG, average daily gain; ADFI, average daily feed intake; V/C, the ratio of villus height to crypt depth; PG1–5, protegrin 1–5; pBD2, porcine beta-defensin 2; TLR-2, toll-like receptor 2; TLR-4, toll-like receptor 4; TNF-α, tumor necrosis factor-α; IL-6, interleukin- 6; IL-8, interleukin- 8

The Spearman correlation analysis between significantly differential intestinal microbes and the performance, morphological injuries indicators, and ileal immune response parameters was shown in Fig. 7.

As showed in Fig. 7A, final body weight and ADG, as well as ADFI, were negatively (*P* < 0.05) associated with Proteobacteria (phylum), γ-Proteobacteria (class), Pseudomonadales (order), Moraxellaceae (family), *Acinetobacter* (genus), unidentified Clostridiales (family), and *unidentified Clostridiales* (genus). Final body weight was also correlated (negatively, *P* < 0.05) with Bacillales (order), Planococcaceae (family), and *Kurthia* (genus), but represented the opposite (*P* < 0.05) associations with Bacteroidetes (phylum), Bacteroidia (class), Bacteroidales (order), and Ruminococcaceae (family). Moreover, ADG was positively (*P* < 0.05) associated with Bacteroidales (order), Ruminococcaceae (family), Lactobacillaceae (family), and *Lactobacillus* (genus), while negatively (*P* < 0.05) associated with *Clostridium disporicum* (species), Bacillales (order), Planococcaceae (family), and *Kurthia* (genus). Additionally, positively (*P* < 0.05) correlations between ADFI and Bacteroidales (order), Ruminococcaceae (family), Bacilli (class), Lactobacillales (order), Lactobacillaceae (family), *Lactobacillus* (genus), and *Lactobacillus_reuteri* (species) were observed, but represented the opposite (*P* < 0.05) correlation with *Clostridium disporicum* (species).

As displayed in Fig.7B, negative (*P* < 0.05) associations between Proteobacteria (phylum), and its aligned bacteria and jejunal villus height, ileal V/C, and ileal microvillus height were observed. Bacteroidetes (phylum) showed positively (*P* < 0.05) correlated with the duodenal villus height, jejunal and ileal microvillus height. Belong to Firmicutes (phylum), the unidentified Clostridiales (family), *unidentified Clostridium* (genus), *Clostridium disporicum* (species), Bacillales (order), Planococcaceae (family), and *Kurthia* (genus) shown positively (*P*<0.05) correlated with villus height, and microvillus height of small intestine sections. The value of V/C in different small intestine sections was all negatively (*P* < 0.05) correlated with Streptococcaceae (family) and Streptococcus (order). In addition, duodenal V/C was negatively (*P* < 0.05) associated with Bacillales (order), Planococcaceae (family), while jejunal V/C showed the same associations with Bacilli (class) and *Lactobacillus amylovorus* (species) as well as ileal V/C with Bacillales (order), Planococcaceae (family), and *Kurthia* (genus). Moreover, microvillus height was positively (*P* < 0.05) associated with Bacteroidetes (phylum), Bacteroidia (class), and Ruminococcaceae (family), but showed the opposite associations (*P* < 0.05) with γ-Proteobacteria (class), Pseudomonadales (order), Moraxellaceae (family), *Acinetobacter* (genus), unidentified Clostridiales (family), *unidentified Clostridium* (genus), *Clostridium disporicum* (species), Bacillales (order), Planococcaceae (family), *Kurthia* (genus), Bacilli (class) and *Lactobacillus amylovorus* (species).

As exhibited in Fig.7C, antimicrobial peptide (*PG1–5*, *pBD-2*) and mucins (mucin 1) were associated (positively, *P* < 0.05) with Proteobacteria (phylum), γ-Proteobacteria (class), Pseudomonadales (order), Moraxellaceae (family), *Acinetobacter* (genus), Streptococcaceae (family) and *Streptococcus* (genus), but showed the opposite correlated (*P* < 0.05) with Bacteroidetes (phylum), Bacteroidia (class). The gene expression of pro-inflammatory cytokine such as *TNF-α* and *IL-6* was positively (*P* < 0.05) associated with Proteobacteria (phylum), γ-Proteobacteria (class), Pseudomonadales (order), Moraxellaceae (family), *Acinetobacter* (genus), and negatively (*P* < 0.05) associated with Bacteroidetes (phylum), Bacteroidia (class). Positively (*P* < 0.05) associations between the protein expression of P65 with Pseudomonadales (order), Moraxellaceae (family), Bacillales (order), Planococcaceae (family), *Kurthia* (genus), Streptococcaceae (family) and Streptococcus (order) were found, but represented the opposite (*P* < 0.05) associations with Bacteroidetes (phylum) and Bacteroidia (class).

## Discussion

Previous studies showed that FI reduction is one of the main adaptations employed to reduce heat production in response to HS [4, 7, 12, 28]. In line with previous results, we found that 7-day constant chronic HS caused hyperpyrexia, drastically ADFI declined (61%), and weight loss. Meanwhile, hypothermia in FI can be explained by calorie restriction which can lower core body temperature in endothermic animals [33].

It is well documented that intestinal epithelial cells under HS experiencing ischemia, hypoxia, inflammation, and oxidative stress can result in morphological injuries such as villus tips desquamation, villus height shorter, and lamina propria exposed [7, 34]. Coincided, morphological injuries in the small intestine were also observed in heat-stressed pigs in the present study. Several studies found that HS can lead to intestinal barrier dysfunction and hyperpermeability by reducing and redistributing tight junction (TJ) proteins [34, 35]. However, in this study, the results indicated a compensatory increase of TJ proteins (ZO-1, Occludin) in the duodenum and ileum along with HSP 70 elevated. HSPs, a class stress-inducible protein, aids in protein folding, ubiquitination, renaturing, and repairing, were found to protect heat-induced intestinal tight junction barrier disruption by up-regulation and reorganization of TJ proteins [36]. It was reported that HS-induced up-regulation of Occludin expression in Caco-2 cells is mediated by activation of heat shock factor-1(HSF-1), which can promote the expression of HSPs [37]. In addition, HS exposure results in the overexpression of HSPs in organs of different species [7, 38–42]. Moreover, HSPs expression in the intestine were considered to be more sensitive to long-term stress than conventional stress markers such as serum concentrations of catecholamines, cortisol, lactate [38], and showed significant associations with intestinal immunity [42].

Increased intestinal permeability caused by HS may result in bacterial translocation and pathogen loads elevated, which can activate numerous bacterial endotoxin-specific recognition receptors in intestinal epithelial cells. Toll-like receptors are a class of classical recognition receptors, such as *TLR-2* and *TLR-4*, which can recognize the damage-associated molecular patterns to produce several pro-inflammatory cytokines to evoke the host immune response during heat stress [15, 17]. In this study, we found that HS up-regulated *TLR2/4* mRNA expression meanwhile elevated the expression of pro-inflammatory cytokines *TNF-α, IL-6, IL-8* as well as *P65*, indicated the activated intestinal immune response under HS, which is consistent with previous report [18]. Epithelial cells can produce mucins and anti-microbial peptides, which can enhance the intestinal epithelial barrier function by selective microbial permeation and directly influencing microbial populations [43, 44]. The increased gene expression of mucins (mucin-1, mucin-2) and anti-microbial peptides (*PG1–5*, *pBD-2*) may be speculated as another compensatory mechanism to endogenously protect the intestinal epithelial barrier. In addition, the results of a feed restriction test suggested that 8-day 80% restricted intake caused 172 genes in immune response pathways upregulated in adipose tissue of weaning pigs [45]. However, the ileal immune response was not affected by PF in the current study. Too much FI reduction (61%) in the present study may cause malnutrition, which would explain the differences.

Firmicutes (phylum) and Bacteroidetes (phylum) have been reported as predominant microbial communities in pig gut microbiota [46, 47]. This agrees with our data, Firmicutes (phylum) and Bacteroidetes (phylum) total comprise 67.33–86.80% in pigs’ gut microbiota. Based on the results of our previous study [28], we prolonged HS duration to 7 days in the current study, and we found that 7-day constant HS also decreased the relative abundance of Bacteroidetes (phylum), and at the same time increased Proteobacteria (phylum) as the second abundant microbe instead of Bacteroidetes (phylum). Unfortunately, opportunistic pathogen *Acinetobacter* (genus), *Kurthia* (genus), and pathogen *Streptococcus* (genus) were observed prevalent in HS. The intensity of host response to HS is presumably decided by heat loads and duration [27], how the gut microbiota is changed by heat loads and duration need further investigations.

Increasing evidences have suggested the crosstalk between gut and host through microbiota [43, 48, 49]. Reduction of FI by HS appears to be affected by appetite-related signals, post-absorptive metabolism, and intestinal function and integrity [3, 50]. A previous study has suggested that microbiota can participate in the regulation of host appetite-related pathways by its metabolites, for example, *Escherichia coli* can activate satiety via its by-product caseinolytic protease (Clp) B, which is an antigen-mimetic of anorexigenic factor α-melanocyte-stimulating hormone(α-MSH) [51]. Associations between FI and alterations in gut microbiota have been analyzed in pigs in our previous study, and we found that FI is positively associated with Bacteroidetes (phylum), Bacteroidia (class), Bacteroidales (order) and Prevotellaceae (family) and negatively associated with Proteobacteria (phylum), γ-Proteobacteria (class), Pseudomonadales (order), Moraxellaceae (family) and *Acinetobacter* (genus) in 24-h acute heat-stressed pigs [28]. Similarly, in the current work, FI was observed positively associated with Bacteroidales (order), and negatively associated with Proteobacteria (phylum), γ-Proteobacteria (class), Pseudomonadales (order), Moraxellaceae (family) and *Acinetobacter* (genus). In heat-stressed ducks, ADG was reported to be negative correlated with Proteobacteria (phylum), and positive associated with Firmicutes (phylum) in the jejunum [25]. Differently, in the present study, ADG showed no significant association with Firmicutes (phylum). We found that sampling location in the gastro-intestine, species, and statistical method would affect above correlation analysis results, therefor bacteria transplantation technique would be a better choice to explore the relationship between the microbiota and phenotypic characteristics.

In this paper, the intestinal morphological injuries indicators showed positive associations with Proteobacteria (phylum) and negatively with Bacteroidetes (phylum). Gut microbiota can influence the host through their by-products, such as short-chain fatty acids (SCFAs), which are not only energy sources but also beneficial to intestinal epithelial integrity [52]. Nevertheless, as one of the main of SCFAs producing bacteria [53], Bacteroidetes (phylum), Bacteroides (class), and Bacteroides (order) are lower in HS in present study. Moreover, butyrate-producing bacteria Ruminococcaceae (family) [54] was also reduced by HS in the current work. Declined of SCFAs producing bacteria means fewer SCFAs, and lead to intestinal epithelial integrity injuries. The results of jejunum microbiota in heat stressed-ducks were partly similar to our results that the morphological injuries were observed positively with the relative abundance of Proteobacteria (phylum) and negatively with Firmicutes (phylum), and this is a possible reason for different species [25]. Moreover, the prevalence of opportunistic pathogen *Acinetobacter* (genus), *Kurthia* (genus), and pathogen *Streptococcus* (genus) in HS caused intestinal inflammation and apoptosis, which may be another reason for morphological injuries.

We found the mucosal immune activation via *TLR4/*nuclear factor kappa-B* (TLR4/NF-κB)* signaling pathway in the ileum by HS. The results of the fecal transplantation experiment also indicated that changes in gut microbial composition promoted by HS could activate the *TLR4/NF-κB* signaling pathway and cause inflammation in the colon of pigs [18]. Moreover, stress induces increased permeability of the gut allowing bacteria translocation and mucosal immune activation, which were reported to alter gut microbial composition in turn [48]. Crosstalk between the microbiota and intestinal immune system is critical in the maintenance of mucosal homeostasis. Accumulation evidences indicated that commensal and pathogenic bacteria and their metabolites participate in the metabolic process [43, 48, 49]. In this work, the results revealed that ileal immune response by HS positively with Proteobacteria (phylum), Streptococcaceae (family), Bacillales (order) and negatively with Bacteroidetes (phylum).

FI reduction caused by HS was thought to a main reason for morphology injuries and alterations in gut microbiota [6, 10, 31]. It is often difficult to discriminate the indirect influence of FI reduction under HS. Therefore, a pair-fed model was utilized in the current study, and we found a separate cluster of PF from TN and HS indicated the difference between PF and HS. Probiotics such as *Lactobacillus* (genus) were reported significantly favored by life-long feed restriction in the intestine of rodents [30]. Conversely, we found that PF decreased Lactobacillales (order) in the current study. Differences in species, growth stage, duration of feed restriction, and amount of FI reduction would explain the discrepancy.

Modifying feed behavior such as feed distribution frequency and size has been reported to affect the performance and metabolic of pigs reared in TN conditions [55]. The limitation of the present study is that the experimental design of PF model just considered the meal size but did not consider the change of feed frequency affected by HS. Fortunately, we found a study by Serviento and colleagues which reported that increased feed provision frequency to 8 times a day did not improve the HS response of pigs [56]. Therefore, further work is urgently needed in the exploration of how to improve the HS response of pigs via feeding management.

## Conclusions

Intestinal morphological injuries and ileal immune response caused by constant chronic HS independent of FI showed close connections with alterations in intestinal microbiota in growing-finishing pigs. This study provided a better understanding of the mechanisms involved in heat stress response, in particular regarding the involved immune response and the epithelial barrier dysfunction. How the gut microbiota alterations under HS affect the host’s gut immunity and the underly mechanism need to be further explored.

## Data Availability

Raw reads of 16S rRNA sequencing in this study were submitted to the GenBank databases under accession number: SRP324970.

## Abbreviations

HS: Heat stress
FI: Feed intake
PF: Pair-fed
ADFI: Average daily feed intake
ADG: Average daily gain
HSP 70: Heat shock protein 70
ZO-1: Zonula occluden-1
TLR-2: Toll-like receptor 2
TLR-4: Toll-like receptor 4
TNF-α: Tumor necrosis factor-α
IL-6: Interleukin- 6
IL-8: Interleukin- 8
PG1–5: Protegrin 1–5
pBD2: Porcine beta-defensin 2
OUT: Operational taxonomic units
SCFAs: Short-chain fatty acids
NF-κB: Nuclear factor kappa-B
